# Multimodal Prediction of Renal Tumor Malignancy From Radiology Reports and Structured Electronic Health Records: Retrospective Cohort Study

**DOI:** 10.2196/84396

**Published:** 2026-05-27

**Authors:** Zhengkang Fan, Renjie Liang, Chengkun Sun, Jinqian Pan, Russell Terry, Jie Xu

**Affiliations:** 1 Department of Heath Outcome and Biomedical Informatics College of Medicine University of Florida Gainesville, FL United States; 2 Department of Urology College of Medicine University of Florida Gainesville, FL United States

**Keywords:** EHR, electronic health records, large language models, LLM, machine learning, ML, multimodal data fusion, renal tumor malignancy prediction

## Abstract

**Background:**

Accurate preoperative prediction of renal tumor malignancy is critical for guiding decisions and reducing overtreatment, as a substantial proportion of renal masses prove benign. Although radiology assessments and structured electronic health record (EHR) data are routinely used, many tumor-specific descriptors remain embedded in free-text radiology reports and are underused due to extraction challenges.

**Objective:**

This study aimed to develop and evaluate a multimodal pipeline that integrates structured EHR variables with natural language processing features from computed tomography (CT) radiology reports, including large language model (LLM)–extracted abnormality characteristics and transformer-based report embeddings, to improve malignancy prediction.

**Methods:**

We conducted a retrospective cohort study using University of Florida Health Integrated Data Repository Observational Medical Outcomes Partnership–mapped EHR data from December 2011 to August 2024. Adults with renal tumors were included if they had longitudinal diagnostic documentation consistent with a renal mass and at least 1 preoperative renal CT report; final benign or malignant status served as the outcome. Structured features included demographics, comorbidities, medications, vital signs, and laboratory measurements. From the recent preindex CT report, an on-premises LLM isolated kidney-specific findings and extracted abnormality characteristics. Four locally deployed LLMs were evaluated against manual annotations of 500 reports. Kidney-specific text was encoded using pretrained biomedical transformer models, including radiology Bidirectional Encoder Representations from Transformers (BERT) variants. We evaluated unimodal baselines and multimodal early, middle, and late fusion strategies. Model development used 5-fold cross-validation within the 80% training partition; each fold-specific model was evaluated on the same independent 20% held-out test set, with performance reported as mean and SD across the 5 held-out test evaluations. The primary metric was area under the receiver operating characteristic curve (AUC).

**Results:**

The final cohort included 967 patients (n=712, 73.6% malignant). In extraction evaluation, Qwen2.5-32B achieved 88.3% overall accuracy with a 100% extraction success rate and was selected for downstream feature generation. Among unimodal models, the structured clinical variable model achieved an AUC of 0.758 (SD 0.012), kidney-specific text with radiology BERT achieved an AUC of 0.746 (SD 0.058), and abnormality characteristics alone achieved an AUC of 0.716 (SD 0.015). Multimodal fusion models achieved higher descriptive performance than unimodal models. Early fusion achieved the highest AUC (mean 0.813, SD 0.008), and *F*_1_-score (mean 0.809, SD 0.030), while late fusion achieved an AUC of 0.805 (SD 0.016). Ablation and interpretability analyses suggested complementary predictive information from structured clinical variables and kidney-specific text embeddings.

**Conclusions:**

Integrating unstructured radiology report text with structured EHR variables achieved higher mean predictive performance than unimodal approaches in descriptive comparisons. Multimodal fusion, particularly early fusion incorporating radiology BERT–derived kidney-specific text embeddings, achieved the strongest discrimination, suggesting potential value of natural language processing–enabled multimodal EHR pipelines for informing preoperative risk stratification.

## Introduction

Kidney cancer (KC) is the seventh most common cancer in the United States, with renal cell carcinoma (RCC) accounting for approximately 90% of cases [1,2]. In 2025, a total of 80,980 new RCC cases were estimated, with over 64.7% occurring in males [3]. Early-stage KC is often asymptomatic, and more than half of cases are detected incidentally during abdominal imaging [4]. While surgical resection, partial or radical nephrectomy, remains the primary treatment, nearly 25% of these small tumors prove to be benign postoperatively [5,6]. These unnecessary surgeries expose patients to potential complications without therapeutic benefit, underscoring the urgent need for improved preoperative risk stratification.

Cross-sectional imaging, particularly computed tomography (CT), plays a central role in the diagnosis and management of renal masses, and many imaging-based models (eg, radiomics or deep learning) have demonstrated promising predictive capabilities. For example, deep learning models applied to preoperative CT imaging have achieved the area under the receiver operating characteristic curve (AUC) of up to 0.87 in differentiating benign from malignant tumors [7-10], and up to approximately 0.86 in predicting RCC subtypes [11]. Prognostic models for Stage, Size, Grade, and Necrosis scores or survival outcomes have also shown concordance indices between 0.75 and 0.84 [12,13]. However, these approaches typically require access to raw imaging data and specialized computational infrastructure, limiting scalability in resource-constrained health care settings.

In contrast, electronic health records (EHRs) are widely available and contain rich, routinely collected data, such as demographics, comorbidities, medication histories, and clinical notes (eg, pathology reports and radiology reports), which can be harnessed to develop predictive models. Structured EHR data captures known RCC risk factors (eg, BMI, smoking history, cardiovascular disease, and diabetes) [14], and has been successfully used in risk stratification for various diseases [15,16]. Thus, incorporating EHR data into renal tumor prediction models offers a scalable, cost-effective alternative to image-based approaches.

While structured EHR data offers important clinical information, approximately 80% of health care information remains unstructured, such as narrative texts, images, and signals [17]. Tumor-specific details (eg, size, texture, and imaging descriptors) are often embedded in radiology reports rather than structured fields. In response, researchers have used natural language processing (NLP) techniques to extract granular tumor characteristics from free-text clinical documentation [18,19]. Rule-based NLP frameworks have demonstrated efficacy in retrieving tumor-related information from unstructured texts [20,21]; however, these approaches typically necessitate labor-intensive development, including manual rule crafting and domain-specific engineering, which constrains their scalability and cross-institutional applicability. In contrast, large language models (LLMs), such as GPT-4 [22] and LLaMA-2 [23], offer improved adaptability and scalability due to their deep contextual understanding acquired through extensive pretraining. Empirical evidence indicates that LLMs can extract structured oncologic information from narrative radiologic and pathologic reports with minimal manual intervention [24-28].

Building upon these advances, recent studies have explored the multimodal integration of structured EHR data with unstructured textual and imaging features to improve predictive modeling, particularly in oncology [29,30]. For example, Xi et al [31] developed a deep learning framework that combined routine magnetic resonance imaging with structured clinical variables to differentiate benign from malignant renal lesions. Similarly, Xu et al [32] integrated clinical data with radiomic features derived from CT to support tumor classification. In parallel, combining NLP-processed clinical narratives with structured data has been shown to boost predictive performance across diverse medical domains [33-35]. Although research specifically targeting renal tumors is still limited, evidence from related applications suggests strong translational potential.

**Figure 1:**
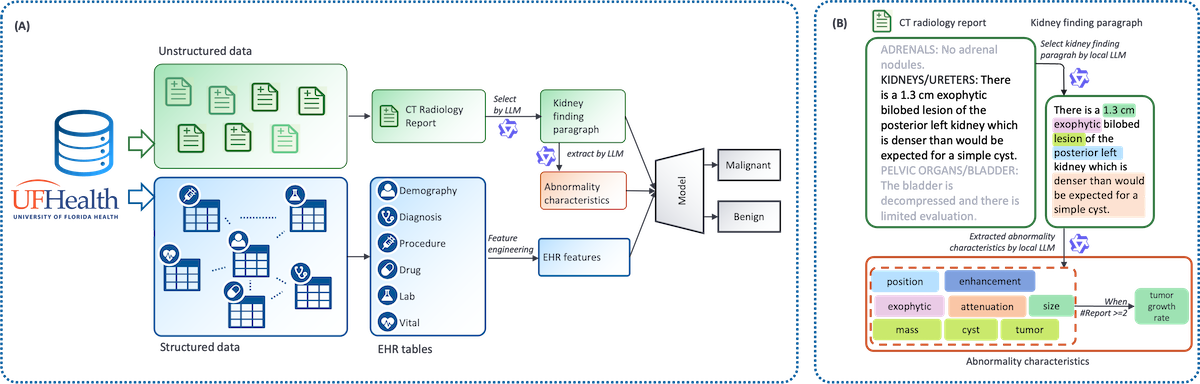
(A) Overview of the proposed multimodal predictive pipeline. (B) Workflow for feature extraction from radiology reports. CT: computed tomography; EHR: electronic health report; LLM: large language model.

In this study, we developed a multimodal pipeline that integrates structured EHR data with features extracted from unstructured radiology reports. An LLM was used to extract abnormality descriptors from free-text narratives, and additional engineered features were derived from tabular EHR data. Each information stream, structured clinical variables, kidney-specific findings, and abnormality characteristics, was encoded via a specialized architecture: Bidirectional Encoder Representations from Transformers (BERT) processed the textual kidney-related observations, while a multilayer perceptron (MLP) handled the structured clinical variables and engineered abnormality characteristics. The resulting embeddings were then combined through a fusion strategy designed to evaluate whether multimodal integration could enhance descriptive model performance for renal tumor prediction. Importantly, integrating structured and unstructured data not only enhances model performance but also enables the creation of more comprehensive patient profiles that could support more individualized assessment and better-informed clinical decisions. An overview of the pipeline is shown in Figure 1A.

## Methods

### Data Sources and Cohort Definition

Data were obtained from the University of Florida Health Integrated Data Repository, which houses its clinical content in the Observational Medical Outcomes Partnership Common Data Model. The structured dataset included demographics, diagnoses, medications, procedures, and laboratory results, while unstructured data comprised CT radiology report narratives. We retrospectively identified over 60,000 patients with renal-related conditions observed from December 2011 to August 2024. The study cohort included patients who met all of the following criteria: (1) had at least 2 distinct renal tumor diagnoses, recorded at different time points, based on *ICD* (*International Classification of Diseases*) codes, with the earliest diagnosis designated as the index date; (2) experienced at most 1 change in tumor classification (benign to malignant or malignant to benign), with the final status serving as the outcome label (patients with multiple reversals were excluded); and (3) had at least 1 renal CT report dated before the index date. A schematic illustrating the temporal ordering of imaging, diagnosis, outcome labeling, and surgical intervention is provided in Figure S9 in Multimedia Appendix 1.

### Outcome Definition and Sensitivity Analyses

Benign vs malignant tumor status was derived from longitudinal EHR diagnosis codes, as pathology reports were not uniformly available. Patients were classified as benign or malignant if they had at least 2 corresponding *ICD* diagnosis codes recorded on distinct dates, with the final observed status used as the outcome label. To reduce label instability, patients with multiple benign-malignant reversals were excluded, and a 1-direction transition rule was applied.

To assess the robustness of the *ICD*-based outcome definition, sensitivity analyses were conducted in patient subsets with higher-specificity reference signals. These included (1) a pathology-confirmed subset identified from unstructured surgical pathology reports and (2) a nephrectomy subset, in which renal tumor diagnosis codes recorded after surgery were used as proxy reference labels. These analyses were used to evaluate the consistency of outcome labeling under more stringent definitions.

### Data Preprocessing

We integrated structured EHR data with CT radiology report analyses to generate three feature sets for modeling: (1) structured EHR variables; (2) kidney-specific findings extracted from radiology reports; and (3) tumor characteristics derived from these findings. The detailed workflow for feature extraction from radiology reports is shown in Figure 1B.

Structured clinical variables were retrieved from tables containing patient demographics, diagnoses, procedures, medications, vital signs, and laboratory results. The observation window extended from the earliest available record up to the index date. Demographic variables (eg, age, sex, and race/ethnicity) were categorized, diagnoses were mapped using PheWAS (Phenome-Wide Association Study) Phecode groupings [36], and medications were aggregated at the ingredient level using Anatomical Therapeutic Chemical classification codes [37]. For vital signs and laboratory tests, the most recent values preceding the index date were selected. For blood pressure, the highest and lowest systolic and diastolic values recorded on the most recent measurement day were used as continuous summary features. Dialysis procedures were encoded as binary indicators. Categorical features were one-hot encoded, and continuous variables were standardized prior to modeling.

To isolate kidney-specific findings, the most recent radiology report within the observation window was selected. Given that abdominal CT reports often include findings for multiple organs, the LLM Qwen2.5-32B [38] was then used to isolate the paragraph exclusively detailing kidney-specific findings, yielding a consolidated narrative for downstream analysis. The model was hosted on premises to ensure patient confidentiality and maintain data integrity.

Abnormality characteristics were extracted from the kidney-specific narratives using an LLM to capture detailed lesion descriptors absent from structured data. Extracted features included abnormality presence, type (eg, cyst, mass, or tumor), lesion size, anatomical position (eg, left kidney or right kidney), exophytic status, CT attenuation, and contrast enhancement. For patients with multiple preindex renal CT examinations, tumor growth rate was calculated using only lesion size measurements obtained prior to the index date, defined as the earliest recorded renal tumor diagnosis. Specifically, when at least 2 preindex imaging studies were available, growth rate was computed as the slope of lesion size change (cm per day) between the earliest and latest available preindex imaging studies.

Missing values in structured EHR variables were handled using modality-specific strategies. Before imputation, laboratory variables with greater than 50% missingness were excluded from the structured clinical feature set. Continuous features (ie, laboratory measurements and vital signs) were imputed using mean values calculated from the full cohort, and categorical variables (ie, diagnosis and medication records) were processed via standard presence-absence encoding, with missing entries encoded as 0 (eg, absence of a diagnosis or medication record). For radiology-derived abnormality characteristics, categorical attributes were treated as “unknown” when not explicitly mentioned and encoded as a separate category. Continuous abnormality variables (eg, lesion size and tumor growth rate) were encoded using a 2-part sentinel representation. For cases where size information was unavailable, or where fewer than 2 preindex longitudinal imaging studies were present to compute a growth rate, the numeric value was imputed with a placeholder of zero. This value was paired with a corresponding binary missingness indicator to ensure the model could mathematically distinguish between missing measurements and true physiologic stability (eg, a measured growth rate of zero). Both the numeric placeholder and the binary indicator were included as independent features in the model input. Kidney-specific text findings were encoded using pretrained biomedical transformer models, producing fixed-length embeddings of 768 dimensions. More details can be found in Table S7 in Multimedia Appendix 1.

### LLM Extraction Evaluation

To evaluate the performance of LLMs in extracting these abnormality characteristics, we manually annotated 500 independent radiology reports to establish a gold-standard dataset. The gold-standard annotations were created by 2 independent nonexpert annotators with biomedical research training, following detailed annotation guidelines developed in consultation with board-certified radiologists. Annotators independently labeled each of the 500 radiology reports, blinded to model outputs. Interrater reliability was assessed using Cohen κ for each abnormality attribute, with disagreements resolved through consensus discussion guided by a radiologist.

For automated extraction, the pipeline identifies kidney-related sentences, extracts lesions with their attributes as structured entries, validates outputs against a predefined schema, and ranks lesions by severity, prioritizing tumors over masses and cysts, and ordering within categories by size. Four locally deployed LLMs (Qwen2.5-7B [38], Qwen2.5-32B [38], LLaMA3-8B [39], and LLaMA3-70B [39]) were evaluated in inference-only mode under institutional data privacy constraints, without any task-specific model training.

To quantify extraction performance, 2 components of the pipeline were assessed. Kidney-specific paragraph retrieval was evaluated by comparing automatically retrieved paragraphs with manually annotated reference paragraphs using the Bilingual Evaluation Understudy-4 (BLEU-4) metric [40], which measures n-gram overlap between predicted and gold-standard text. Performance was summarized as the average of the BLEU-4 score and the percentage of samples achieving a BLEU-4 score greater than 0.5 across reports. A BLEU-4 score above 0.5 indicates good alignment between predicted and reference text in terms of vocabulary, phrase structure, and semantic content, and was considered acceptable for practical downstream use. Abnormality characteristic extraction was evaluated by computing per-attribute accuracy based on an exact match between model outputs and gold-standard annotations. All performance metrics were calculated on the same set of 500 annotated radiology reports.

### Modeling Approach and Fusion Strategies

#### Overview

**Figure 2:**
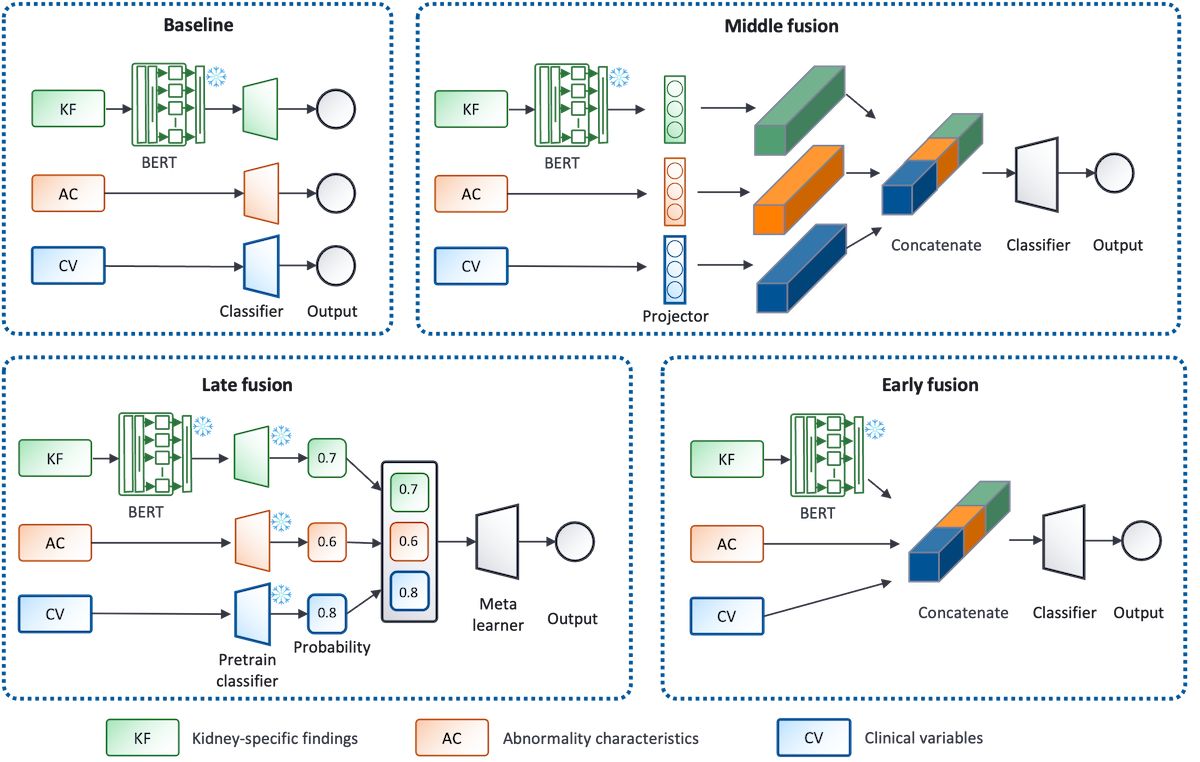
Detailed architecture of the multimodal prediction model.

To integrate the 3 feature modalities, we implemented and compared 3 fusion strategies: early, intermediate (middle), and late fusion, alongside unimodal baseline models (Figure 2).

#### Baseline Models

We independently evaluated structured clinical variables and radiology-derived tumor attributes using a range of machine learning algorithms, including least absolute shrinkage and selection operator regression [41], logistic regression [42], MLP [43], random forest (RF) [44], support vector machine (SVM) [45], and extreme gradient boosting [46]. For narrative kidney-specific findings, 4 pretrained biomedical transformer models (radiology BERT [RadBERT] [47], BERT pretrained on clinical text [ClinicalBERT] [48], biomedical BERT [BioBERT] [49], and BERT pretrained on PubMed text [PubMedBERT] [50]) were used to generate text embeddings. For structured clinical variables and engineered lesion features, model performance was assessed under different resampling strategies (no resampling, random undersampling, random oversampling) to address class imbalance. For the text modality, the 4 transformer models were directly compared to identify the most effective baseline embedding approach.

#### Early Fusion

This approach integrates all 3 modalities at the input stage. Kidney-specific findings were first encoded into dense vectors using the best-performing pretrained transformer model selected from the 4 candidates described above. These embeddings were then concatenated with structured clinical variables and radiology-derived tumor attributes to form a combined feature vector, which was classified using a shallow 2-layer MLP. To mitigate overfitting in this high-dimensional space, we used dropout (0.1) after each hidden layer and Adam with decoupled weight decay (AdamW) optimization with weight decay (0.01; excluding bias and LayerNorm weights), providing L2 regularization of trainable parameters.

#### Middle Fusion

In this architecture, each modality was independently projected into a latent embedding space before being merged and passed through a 2-layer MLP classifier. To evaluate the effect of embedding choice, early and intermediate fusion experiments were repeated using each of the 4 BERT variants. Specifically, modality-specific inputs were compressed into lower-dimensional representations through dedicated projection layers with rectified linear unit activation and dropout (0.1) prior to fusion, thereby reducing dimensionality and mitigating overfitting risk. Training was performed using AdamW optimization with weight decay (0.01; excluding bias and LayerNorm weights) to ensure consistent L2 regularization.

#### Late Fusion

Predicted probabilities from the unimodal base models were combined using a logistic-regression meta-learner [42]. To avoid bias from in-sample predictions, an out-of-fold (OOF) stacking strategy was applied. After splitting the dataset into an 80% training set and a 20% held-out test set, 5-fold cross-validation was conducted within the training set. Base models generated probabilities only for their validation folds, producing OOF predictions. For each fold, the meta-learner was trained using the concatenated OOF probabilities from the other folds and evaluated on the held-out test set using the corresponding fold-specific test probabilities. This procedure ensured that the meta-learner was trained only on predictions from samples not used to fit the base models.

### Model Validation and Performance Metrics

Model performance was assessed using a stratified 80/20 train-test split. The 80% training partition was used for model development with 5-fold cross-validation. In each iteration, 1 fold was used for internal validation, and the remaining 4 folds were used for training. The resulting 5-fold–specific models were each evaluated on the same independent 20% held-out test set, which was not used for training, validation, resampling, or hyperparameter tuning. For the machine learning classifiers, hyperparameters were tuned via grid search, with model selection guided by the minimization of negative log-loss on the validation folds. In parallel, deep neural networks were trained using the AdamW optimizer and a linear learning rate scheduler with an initial warm-up phase, with cross-entropy loss serving as the selection criterion on validation subsets.

The primary evaluation metric was the AUC. Secondary metrics included overall accuracy, balanced accuracy, sensitivity (recall for the malignant class), specificity, precision (positive predictive value), and *F*_1_-score, all reported as the mean and SD across 5-fold–specific models evaluated on the independent held-out test set. Balanced accuracy was included to provide a more representative assessment of the model’s discriminative performance beyond class prevalence by equally weighting sensitivity and specificity in the presence of class imbalance. Decision thresholds for probability-based models were determined by maximizing the Youden J statistic. To address class imbalance, we compared 3 approaches: no resampling, random undersampling, and random oversampling. To prevent data leakage, all resampling procedures were performed strictly within the training partition of each cross-validation fold; validation folds and the held-out test set were preserved in their original class distributions.

### Interpretability Analyses

We assessed the contribution of each modality to the best-performing multimodal fusion model using 2 complementary approaches.

#### Kidney-Specific Findings (Text Modality)

We assessed the contribution of individual tokens from the text modality by applying Captum’s layer-integrated gradients to the BERT embedding layer of our best-performing multimodal fusion model. First, we encapsulated the pretrained model in a wrapper that returns class-probability outputs via a SoftMax over logits. Using the wrapped model and specifying the BERT word-embedding layer as the attribution target, we computed layer-integrated gradients for each test sample with 50 interpolation steps. For each test sample, we summed the absolute attributions over the embedding dimension to yield a scalar score per token, then merged WordPiece subtokens into full words by concatenating “##” continuations and averaging their scores. We filtered out trivial tokens (length ≤2), retained the top 15 highest-scoring words per sample, and accumulated counts and total scores across the dataset. Dividing total attribution by occurrence count produced an average-importance score for each word. To improve robustness, this analysis was performed for the best model from each of the 5 cross-validation folds; keyword importance scores were then averaged across folds, and the final top-15 keywords were selected based on the aggregated importance ranking.

#### Abnormality Characteristics and Clinical Variables Feature Analysis

To disentangle and measure the respective contributions of abnormality characteristics and clinical variables to the fusion model’s outputs, we developed 2 dedicated input filters that retain only 1 modality at a time while nullifying all others and applied a gradient-based Shapley value explainer with a randomly selected reference set of up to 100 background samples. For each held-out sample, we computed local attributions for the positive class and derived the mean absolute contribution of each feature across the test cohort, thereby identifying the 15 most influential attributes. These results were summarized using a concise bar chart of mean absolute attributions and a beeswarm plot illustrating the polarity and variability of each feature’s contribution.

### Ethical Considerations

The study has been approved, and the requirement to obtain any informed consent has been waived by the University of Florida Institutional Review Board (protocol number IRB202100401). The research does not involve greater than minimal risk for participation. Analyses only involve the secondary analysis of data that are either limited data sets or deidentified. Our research team has no direct contact with human subjects. All methods were carried out in accordance with relevant guidelines and regulations.

## Results

### Cohort Characteristics

A total of 967 patients with renal tumors met the inclusion criteria. The mean age at initial diagnosis, whether benign or malignant, was 69 (SD 12.93) years, with approximately 60% of the cohort being male. Among these patients, 712 (73.6%) were diagnosed with malignant tumors. Comorbidities were common: over 90% (874/967) had disorders of the kidney and ureters, and 74% (719/967) had a documented history of hypertension. Smoking was reported in 15.51% (150/967) of patients, and the mean BMI was 30.07 (SD 7.08). Detailed baseline characteristics and comorbidity profiles are summarized in Table S1 in Multimedia Appendix 1. The median observation period was 34.43 (IQR 8.53-73.92) months. Surgical intervention was performed in 401 patients (41.47% of the cohort). Among these, 294 (30.40%) patients underwent partial nephrectomy and 250 (25.85%) patients underwent radical nephrectomy; 143 (14.79%) patients received both procedures. Tumor growth rate was available for 22.2% (215/967) of patients, reflecting those with at least 2 preindex surveillance CT examinations; the remaining cases were treated as missing for this feature.

### Sensitivity Analyses for Outcome Definition

Surgical pathology reports containing renal tumor–related diagnostic statements were available for 89 patients (from 97 reports), of whom 81 (91.0%) were classified as malignant based on pathology text. In this pathology-confirmed subset, the primary *ICD*-based outcome labels were fully concordant with pathology-derived malignancy status. In addition, 401 patients underwent nephrectomy, and among the 383 with postprocedure renal tumor diagnosis codes, 347 (90.6%) were malignant; outcome labels derived from *ICD* codes were fully concordant with postnephrectomy diagnostic coding.

### LLM Extraction Evaluation

Interrater reliability for the manually annotated gold-standard dataset was substantial to near-perfect across abnormality attributes (Cohen κ=0.95-1.00) (Table S10 in Multimedia Appendix 1). Table 1 summarizes the performance of LLMs in kidney-specific paragraph retrieval and abnormality characteristic extraction from radiology reports. As shown in Table 1, Qwen2.5-32B and LLaMA3-70B demonstrated comparable performance in abnormality characteristic extraction. For kidney-specific paragraph retrieval, both models achieved high BLEU-4 scores, with more than 95% (475/500) of reports exceeding a BLEU-4 threshold of 0.5, indicating accurate identification of the relevant anatomical sections. However, accuracy alone does not adequately reflect model usability. Qwen2.5-7B and LLaMA3-8B failed to extract 10.4% (52/500) and 18.2% (91/500) cases, respectively, despite repeated attempts. Additionally, the standard LLaMA3 release was limited by its 8192-token context window, which hindered few-shot prompting with multiple examples. In contrast, Qwen2.5-32B demonstrated more reliable kidney-specific paragraph retrieval across long, heterogeneous radiology reports and supported stable local deployment under institutional data-privacy constraints. Considering paragraph retrieval fidelity, abnormality extraction accuracy, and robustness, Qwen2.5-32B was selected as the primary model for downstream lesion feature extraction.

**Table 1:** Performance comparison of large language models for kidney-specific paragraph retrieval and abnormality characteristic extraction from radiology reports.

			Qwen2.5-7B	Qwen2.5-32B		LLaMA3-8B		LLaMA3-70B
**Kidney-specific findings**								
	Average BLEU^a^-4 score	0.615		0.913	0.878		0.929	
	BLEU-4 ≥0.5, n (%)	332 (66.4)		475 (95)	466 (93.2)		482 (96.4)	
**Abnormality characteristics^b^, %**								
	Abnormality presence	76.3		88.4	76.5		84.6	
	Abnormality category	60.4		83.5	60.6		81.2	
	Position	52.0		81.8	51.8		79.2	
	Size (cm)	53.6		89.1	76.2		78.1	
	Exophytic	94.6		100.0	94.4		100.0	
	Attenuation	46.9		85.6	47.2		86.2	
	Enhancement	41.5		89.4	41.3		88.6	
	Overall accuracy	60.8		88.3	64.0		85.4	
	Extraction success rate	89.6		100	81.8		100	

^a^BLEU: Bilingual Evaluation Understudy-4.

^b^Abnormality characteristics included abnormality presence, type (eg, cyst, mass, or tumor), lesion size, anatomical position (eg, left kidney or right kidney), exophytic status, computed tomography attenuation, and contrast enhancement.

### Modeling Results

The consolidated performance results for all modeling approaches are presented in Table 2. Table 2 reports performance on the independent 20% held-out test set. Values are summarized as mean and SD across 5-fold–specific models, each trained during cross-validation within the 80% training partition and then evaluated on the same held-out test set. In the unimodal analyses, the SVM model applied to structured clinical variables achieved the highest descriptive AUC among the 6 traditional algorithms, achieving an AUC of 0.758 and a balanced accuracy of 0.717, whereas the RF model achieved the highest *F*_1_-score (0.833). For the engineered abnormality characteristics, logistic regression led the same set of models with an AUC of 0.716 and an *F*_1_-score of 0.766. Ablation studies comparing undersampling, oversampling, and no-sampling strategies are provided in Table S2 in Multimedia Appendix 1 for the clinical variable models and Table S3 in Multimedia Appendix 1 for the LLM-engineered abnormality characteristic models. Among the 4 medically pretrained BERT variants evaluated on kidney-specific report findings, RadBERT achieved the highest descriptive performance, yielding an AUC of 0.746 and an *F*_1_-score of 0.719. In addition to AUC and *F*_1_-score, balanced accuracy was reported for all models to account for class imbalance (Table 2). Among fusion models, early fusion achieved the highest balanced accuracy (mean 0.775, SD 0.010), followed by late fusion (mean 0.749, SD 0.013), indicating consistent performance across malignant and benign classes rather than dominance by the majority malignant class.

**Table 2:** Performance comparison (mean and SD) of unimodal and fusion models for renal tumor prediction.

Model		Overall accuracy	Balanced accuracy	Sensitivity	Specificity	Precision	*F*_1_-score	AUC^a^
**Baseline model (clinical variables), mean (SD)**								
	LASSO^b^ (OS^c^)	0.729 (0.026)	0.706 (0.003)	0.754 (0.053)	0.659 (0.051)	0.862 (0.009)	0.803 (0.026)	0.750 (0.006)
	LR^d^ (US^e^)	0.674 (0.047)	0.707 (0.034)	0.638 (0.066)	0.776 (0.041)	0.889 (0.018)	0.741 (0.049)	0.728 (0.043)
	MLP^f^ (NS^g^)	0.618 (0.200)	0.661 (0.090)	0.569 (0.322)	0.753 (0.145)	0.693 (0.388)	0.624 (0.350)	0.670 (0.151)
	RF^h^ (US)	0.761 (0.032)	0.710 (0.013)	0.817 (0.058)	0.604 (0.047)	0.853 (0.008)	0.833 (0.029)	0.754 (0.006)
	SVM^i^ (US)	0.712 (0.038)	0.717 (0.016)	0.708 (0.072)	0.725 (0.071)	0.880 (0.019)	0.782 (0.037)	0.758 (0.012)
	XGBoost^j^ (US)	0.685 (0.063)	0.707 (0.037)	0.660 (0.110)	0.753 (0.105)	0.886 (0.038)	0.751 (0.065)	0.725 (0.044)
**Baseline model (abnormality characteristics), mean (SD)**								
	LASSO (OS)	0.667 (0.048)	0.680 (0.019)	0.653 (0.115)	0.706 (0.144)	0.868 (0.036)	0.739 (0.060)	0.711 (0.018)
	LR (OS)	0.691 (0.040)	0.684 (0.010)	0.698 (0.094)	0.671 (0.114)	0.860 (0.028)	0.766 (0.048)	0.716 (0.015)
	MLP (NS)	0.682 (0.028)	0.677 (0.030)	0.688 (0.085)	0.667 (0.139)	0.857 (0.033)	0.760 (0.037)	0.697 (0.022)
	RF (NS)	0.642 (0.020)	0.677 (0.020)	0.604 (0.032)	0.749 (0.047)	0.871 (0.018)	0.713 (0.021)	0.670 (0.007)
	SVM (OS)	0.692 (0.023)	0.679 (0.007)	0.706 (0.049)	0.651 (0.053)	0.851 (0.012)	0.771 (0.026)	0.688 (0.018)
	XGBoost (NS)	0.634 (0.065)	0.655 (0.021)	0.611 (0.134)	0.698 (0.138)	0.857 (0.036)	0.704 (0.087)	0.663 (0.039)
**Baseline model (kidney-specific findings), mean (SD)**								
	RadBERT^k^	0.665 (0.081)	0.724 (0.045)	0.600 (0.124)	0.847 (0.051)	0.917 (0.015)	0.719 (0.100)	0.746 (0.058)
	ClinicalBERT^l^	0.668 (0.044)	0.683 (0.039)	0.652 (0.069)	0.714 (0.086)	0.866 (0.031)	0.742 (0.045)	0.723 (0.054)
	BioBERT^m^	0.659 (0.073)	0.634 (0.053)	0.687 (0.121)	0.580 (0.124)	0.822 (0.033)	0.743 (0.079)	0.626 (0.068)
	PubMedBERT^n^	0.602 (0.120)	0.572 (0.042)	0.635 (0.291)	0.510 (0.364)	0.813 (0.075)	0.671 (0.165)	0.547 (0.076)
**Fusion model (all 3 modalities), mean (SD)**								
	Early fusion	0.750 (0.029)	0.775 (0.010)	0.722 (0.062)	0.828 (0.069)	0.924 (0.021)	0.809 (0.030)	0.813 (0.008)
	Middle fusion	0.726 (0.028)	0.744 (0.018)	0.705 (0.040)	0.784 (0.016)	0.902 (0.006)	0.791 (0.026)	0.782 (0.011)
	Late fusion	0.736 (0.021)	0.749 (0.013)	0.722 (0.053)	0.776 (0.072)	0.902 (0.022)	0.800 (0.025)	0.805 (0.016)

^a^AUC: area under the receiver operating characteristic curve.

^b^LASSO: least absolute shrinkage and selection operator.

^c^OS: oversampling.

^d^LR: logistic regression.

^e^US: undersampling.

^f^MLP: multilayer perceptron.

^g^NS: no sampling.

^h^RF: random forest.

^i^SVM: support vector machine.

^j^XGBoost: extreme gradient boosting.

^k^RadBERT: radiology BERT.

^l^ClinicalBERT: BERT pretrained on clinical text.

^m^BioBERT: biomedical BERT.

^n^PubMedBERT: BERT pretrained on PubMed text.

Variability across cross-validation folds was observed in some configurations using ClinicalBERT and BioBERT embeddings. Because pretrained transformer embeddings are deterministic for fixed inputs, this variability reflects sensitivity of the downstream fusion classifier to specific training-validation splits in a high-dimensional setting rather than the instability of the embedding models themselves. For example, ClinicalBERT-based early fusion exhibited higher fold-level variance (overall accuracy: mean 0.661, SD 0.226; Table S4 in Multimedia Appendix 1), and BioBERT exhibited variability in middle fusion performance (*F*_1_-score: mean 0.608, SD 0.344; Table S5 in Multimedia Appendix 1), indicating inconsistent fold-level behavior. In contrast, RadBERT-based models demonstrated notably more stable performance across both early and middle fusion strategies.

Multimodal integration was associated with higher descriptive predictive discrimination than unimodal modeling. The middle fusion framework achieved an AUC of 0.782, which was higher than the best unimodal baseline AUC, while the early fusion configuration reached the highest AUC of 0.813 and the highest *F*_1_-score of 0.809. The late fusion strategy, which combined predicted probabilities from top-performing unimodal models through a meta-learner, yielded a competitive AUC of 0.805, with precision and sensitivity values of 0.902 and 0.722, respectively. These results suggest that integrating structured and unstructured data modalities may provide complementary information for renal tumor malignancy prediction.

### Ablation Studies

To quantify the contribution of each data modality, we performed an ablation analysis on each fusion architecture by systematically omitting one modality and measuring the resulting impact on model performance. For the 3 modalities, all pairwise combinations were evaluated and their metrics compared against those obtained using the full 3-modality setup. Detailed results are presented in Table 3. Mean (SD) of each evaluation metric across 5-fold cross-validation is reported. Balanced accuracy was also reported in ablation analyses (Table 3) to ensure that observed performance differences across modality combinations were not driven by class prevalence effects. Input modalities include kidney-specific findings, abnormality characteristics, and clinical variables. Because RadBERT achieved the best overall performance, all ablation results reported in Table 3 are based on models incorporating RadBERT (ie, late fusion combining RadBERT with RF). Ablation analyses for the other 3 BERT variants are provided in Table S4 in Multimedia Appendix 1 (ie, early fusion missing-modality) and Table S5 in Multimedia Appendix 1 (ie, middle fusion missing-modality).

**Table 3:** Ablation study results: effect of excluding individual modalities on fusion model performance.

Modality					overall accuracy		Balanced accuracy		Sensitivity		Specificity		Precision		*F*_1_-score		AUC^a^		
**Early fusion, mean (SD)**																			
	AC^b^ + CV^c^			0.763 (0.052)		0.743 (0.010)		0.785 (0.108)		0.701 (0.108)		0.884 (0.027)		0.827 (0.051)		0.779 (0.010)			
	KF^d^ + CV			0.749 (0.046)		0.759 (0.030)		0.738 (0.087)		0.780 (0.097)		0.907 (0.032)		0.811 (0.049)		0.796 (0.030)			
	KF + AC			0.687 (0.041)		0.727 (0.037)		0.642 (0.048)		0.812 (0.041)		0.905 (0.023)		0.751 (0.038)		0.755 (0.061)			
	KF + AC + CV			0.750 (0.029)		0.775 (0.010)		0.722 (0.062)		0.828 (0.069)		0.924 (0.021)		0.809 (0.030)		0.813 (0.008)			
**Middle fusion, mean (SD)**																			
		AC + CV		0.715 (0.060)		0.698 (0.030)		0.734 (0.118)		0.662 (0.124)		0.862 (0.027)		0.788 (0.063)		0.731 (0.019)			
		KF + CV		0.699 (0.069)		0.730 (0.059)		0.664 (0.083)		0.796 (0.045)		0.899 (0.032)		0.763 (0.066)		0.749 (0.074)			
		KF + AC		0.670 (0.056)		0.711 (0.043)		0.625 (0.081)		0.796 (0.069)		0.897 (0.033)		0.734 (0.058)		0.747 (0.052)			
		KF + AC + CV		0.726 (0.028)		0.744 (0.018)		0.705 (0.040)		0.784 (0.016)		0.902 (0.006)		0.791 (0.026)		0.782 (0.011)			
**Late fusion, mean (SD)**																			
			AC + CV	0.677 (0.038)		0.704 (0.025)		0.648 (0.091)		0.761 (0.125)		0.888 (0.035)		0.745 (0.046)		0.752 (0.021)			
			KF + CV	0.764 (0.025)		0.752 (0.032)		0.778 (0.067)		0.725 (0.119)		0.892 (0.036)		0.828 (0.027)		0.794 (0.026)			
			KF + AC	0.692 (0.040)		0.722 (0.026)		0.659 (0.055)		0.784 (0.014)		0.895 (0.009)		0.758 (0.040)		0.758 (0.037)			
			KF + AC + CV	0.736 (0.021)		0.749 (0.013)		0.722 (0.053)		0.776 (0.072)		0.902 (0.022)		0.800 (0.025)		0.805 (0.016)			

^a^AUC: area under the receiver operating characteristic curve.

^b^AC: abnormality characteristics.

^c^CV: clinical variables.

^d^KF: kidney-specific findings.

In late fusion experiments, all 24 combinations of 6 traditional machine learning classifiers and 4 pretrained BERT models were evaluated. Among these, RadBERT paired with RF emerged as the top-performing configuration (see Table S6 in Multimedia Appendix 1).

Omitting kidney-specific findings led to the greatest performance decline in both middle and late fusion configurations. In contrast, within the early fusion scheme, excluding clinical variables caused the most pronounced drop. Removal of abnormality characteristics had only a modest effect across all 3 fusion strategies, with early and late fusion approaches exhibiting robustness to their absence.

### Interpretability Analyses on Fusion Model

#### Kidney-Specific Findings: Visualization and Interpretation

The interpretability analysis of the text modality revealed distinctive linguistic patterns associated with malignancy predictions. The average contribution scores, computed using layer-integrated gradients, were visualized as a ranked horizontal bar plot (Figure S11 in Multimedia Appendix 1). Each bar represents a token, and bar length corresponds to the token’s mean attribution score toward the model’s positive-class predictions (larger values indicate stronger positive contribution). Representative high-impact terms from the kidney-specific findings notes are listed in Table 4. Tokens such as “greater,” “irregularly,” and “huge” exhibited the highest attribution scores, indicating a strong association with malignancy classification in the multimodal fusion framework.

**Table 4:** Representative words from the kidney-specific findings notes.

Ground truth	Prediction	Kidney-specific findings
Malignant	Malignant	There is a 2.3 cm exophytic lesion on the inferior pole of the *right kidney*, with an attenuation *greater* than expected for simple cyst.
Malignant	Malignant	Within the right kidney posteriorly, there is an *irregularly* enhancing mass measuring approximately 2.7 x 2.7 cm.
Malignant	Malignant	*Huge* left renal mass is again partially *observed*.
Benign	Benign	There are multiple *unchanged* bilateral renal *simple* cysts.

#### Abnormality Characteristics and Clinical Variables Feature Analysis

**Figure 3:**
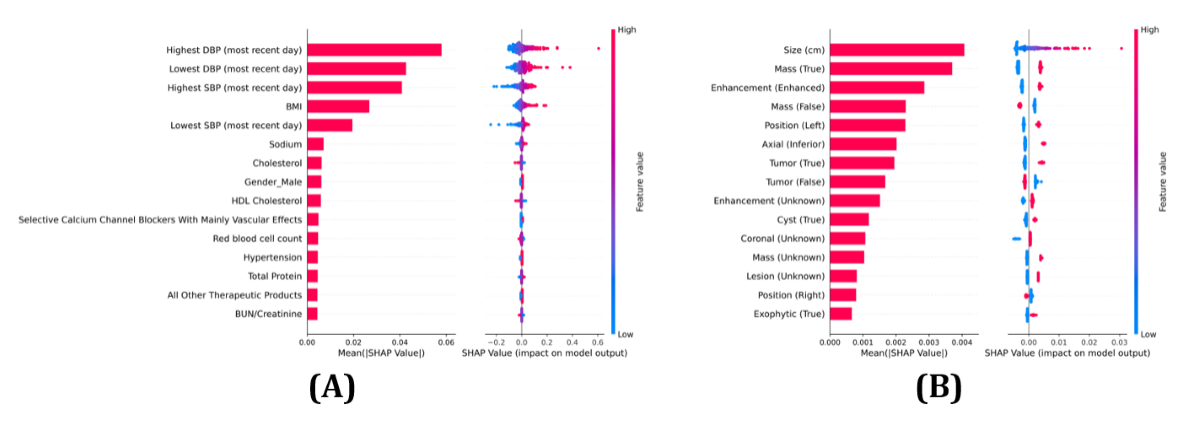
Shapley additive explanations (SHAP) summary plot showing (A) the top 15 clinical variables and (B) the top 15 abnormality characteristics contributing to model prediction. DBP: diastolic blood pressure; HDL: high-density lipoprotein; SBP: systolic blood pressure.

For the structured clinical variables, feature importance rankings derived from gradient-based Shapley values (Figure 3A) indicated that diastolic and systolic blood pressures, BMI, along with serum sodium, were the most influential predictors. Among the abnormality characteristics, lesion size demonstrated the highest importance (Figure 3B), followed by the presence of a mass, with enhancement patterns furnishing critical discriminative information for differentiating benign from malignant lesions.

## Discussion

### Principal Results

Among the unimodal approaches, structured clinical variables showed the highest descriptive predictive performance. Specifically, an SVM classifier with undersampling achieved an AUC of 0.758 (Table 2; Table S2 in Multimedia Appendix 1). By comparison, logistic regression on abnormality characteristics, enhanced via oversampling, yielded an AUC of 0.716 (Table S3 in Multimedia Appendix 1), likely limited by extraction fidelity and feature granularity. Text embeddings from complete paragraph-level kidney-specific findings slightly outperformed the abnormality descriptors, achieving an AUC of 0.746 (Table 2) when encoded with RadBERT [47] (initialized from BioBERT [49] and fine-tuned on radiology reports). PubMedBERT [50], pretrained from scratch on PubMed abstracts and full-text articles, showed comparatively lower performance, likely due to its limited alignment with radiology-report semantics. All 3 multimodal fusion strategies demonstrated higher descriptive AUC values than the unimodal models (Table 2). Early fusion, concatenating feature representations from each modality prior to classification, produced the highest AUC (0.813) and *F*_1_-score (0.809), potentially because it preserves complementary information across modalities. Middle fusion, which first projects each modality into a lower-dimensional latent space, performed slightly worse (AUC 0.782; *F*_1_-score=0.791), possibly due to information loss during dimensionality reduction. Late fusion remained competitive, achieving an AUC of 0.805 and an *F*_1_-score of 0.800, but did not surpass early fusion on any major metric. Overall, these findings indicate that early integration of multimodal information achieved the highest descriptive performance in this study, while late fusion still offered stable and competitive performance through an ensemble-based combination of unimodal predictors.

Although AUC values in the range of 0.8 are insufficient to support automated clinical decision-making, they may offer incremental value for preoperative risk stratification. For patients with indeterminate renal masses, a multimodal model could complement radiologic interpretation by providing an additional quantitative estimate of malignancy risk to inform multidisciplinary discussion and clinical judgment. Such estimates are not intended to direct specific interventions but rather to contextualize imaging findings and patient characteristics within existing diagnostic workflows. Higher specificity, if confirmed in future validation studies, could potentially help reduce unnecessary interventions for benign lesions, an ongoing challenge in renal mass management. Overall, incremental gains in predictive performance may translate into practical support for clinical risk assessment when used alongside established evaluation processes.

### Modality Contributions From Ablation Studies

To assess the contribution of each modality within the fusion architectures, we performed systematic ablation experiments. In the early fusion model, omitting structured clinical variables was associated with the largest decrease in AUC, from 0.813 to 0.755, indicating that clinical variables contributed the strongest predictive signal in this configuration. Removing kidney-specific findings led to a smaller decline in AUC to 0.779, whereas excluding radiologic abnormality characteristics had only a modest effect, with the AUC remaining 0.796. In the middle fusion model, the pattern differed slightly: omitting kidney-specific findings caused the greatest reduction in AUC, from 0.782 to 0.731, while excluding abnormality characteristics or clinical variables resulted in smaller decreases, to 0.749 and 0.747, respectively. In the late fusion model, removing kidney-specific findings caused the largest reduction in AUC, from 0.805 to 0.752, closely followed by omission of clinical variables, which reduced the AUC to 0.758; excluding abnormality characteristics resulted in only a modest decline, with the AUC remaining 0.794. Overall, these ablation results suggest that structured clinical variables were the dominant source of predictive information in the early fusion scheme, whereas kidney-specific text findings contributed most strongly in the middle and late fusion models. Across all 3 fusion strategies, removal of abnormality characteristics produced the smallest or among the smallest performance changes, suggesting that part of the radiologic information captured by these handcrafted features may already be represented in the kidney-specific text embeddings.

### Interpretability Insights

Furthermore, we conducted an interpretability analysis on the optimal early fusion model by applying Captum’s layer-integrated gradients to the kidney-specific text and Shapley additive explanations (SHAP) values to the abnormality characteristics and clinical variables. As illustrated in the horizontal bar plot visualization (Figure S11 in Multimedia Appendix 1) and exemplified in Table 4, tokens such as “huge,” “irregularly,” and “greater” attained relatively high integrated gradients attributions, suggesting a stronger contribution to malignancy predictions. This observation is in line with prior imaging research reporting that irregular tumor margins are associated with higher-grade clear cell RCC [51].

Quantitative SHAP analysis of clinical variables (Figure 3A) showed that higher systolic blood pressure, diastolic blood pressure, and BMI were associated with increased predicted probabilities of malignancy. These associations are consistent with previous epidemiological studies: individuals with diastolic pressure ≥100 mm Hg face more than a twofold higher risk of RCC compared to those with <80 mm Hg [52], and a dose-response meta-analysis demonstrates that each 1 kg/m^2^ increase in BMI is linked to an approximately 6% higher incidence of KC [53]. For abnormality characteristics (Figure 3B), SHAP analysis identified tumor size as the most influential imaging feature, with contrast‐enhancement also contributing meaningfully to the model’s predictions. This finding aligns with prior evidence showing that each 1 cm increase in tumor diameter is associated with a ~30% higher likelihood of malignancy (effect size=1.3, 95% CI 1.22-1.43 per cm) [54]. Furthermore, established imaging guidelines indicate that postcontrast attenuation gains greater than 15 Hounsfield units may aid in distinguishing malignant renal lesions [55]. Collectively, these interpretability results suggest that our multimodal fusion framework captures and integrates clinically validated markers across multiple modalities.

### Limitations

Despite these findings, several limitations should be acknowledged. First, this was a single-institution study, and no external validation was performed. Consequently, model performance may reflect institution-specific characteristics, including documentation practices, patient populations, and radiology reporting styles unique to our health care system. This is particularly relevant for NLP-based feature extraction, which can be sensitive to local language conventions. Second, the retrospective use of EHR data introduces inherent biases related to incomplete or inaccurately recorded information. Mean imputation was applied strictly to continuous variables, specifically laboratory measurements and vital signs. To reduce instability from extreme sparsity, laboratory variables with greater than 50% missingness were excluded before imputation. However, some retained variables, including cholesterol and high-density lipoprotein cholesterol, had missingness rates close to this threshold and remained subject to mean imputation. The missingness percentages for these variables are reported in Table S8 in Multimedia Appendix 1 to provide transparency regarding the extent of missing data. We acknowledge that this simplified strategy may attenuate physiological variance and fail to preserve correlations between related clinical measures, such as metabolic markers, potentially affecting model calibration, particularly for non–tree-based models used in the fusion strategies. In addition, continuous structured variables were imputed and standardized using cohort-level parameters rather than parameters estimated separately within each cross-validation training fold, which may introduce limited information leakage. Third, ground-truth tumor labels were not uniformly confirmed by surgical pathology. For the unoperated majority of the cohort, outcome labels were derived from longitudinal clinical diagnosis codes that may reflect radiologic interpretation rather than definitive tumor biology. This introduces a risk of diagnostic circularity, where the imaging features used for prediction overlap with those informing clinical labeling. While we mitigated this by requiring repeated concordant codes on distinct dates and performing sensitivity analyses on the pathology-confirmed subset, residual misclassification and circularity may persist. To further assess potential temporal leakage from treatment-related terminology, we conducted a temporal audit of all radiology reports used for feature extraction. Among 1338 reports, only 17 (1.27%) contained the term “surgically,” and none of these patients had nephrectomy or other renal procedural codes recorded prior to the index date, suggesting minimal risk of leakage from completed interventions. However, because explicit diagnostic terminology was not systematically removed from the radiology text, some NLP-derived features may partially reflect radiologists’ diagnostic impressions rather than purely morphological descriptors, introducing a potential source of label leakage in the text-derived features. Although the updated interpretability analysis primarily highlighted descriptive terms such as “greater,” “irregularly,” and “huge,” the broader possibility of diagnostic-language leakage remains a limitation of the current approach. Finally, the LLM-based extraction pipeline incurs nontrivial computational requirements. While models were used in inference-only mode, larger architectures like Qwen2.5-32B require high-memory GPU infrastructure for local deployment. Because processing a single radiology report requires several seconds in batch mode, the proposed framework is currently best suited for asynchronous deployment within institutional analytics pipelines rather than real-time clinical use in resource-constrained environments.

### Future Work

Future work should focus on validating this multimodal approach across multiple health care institutions using larger and more diverse patient populations to ensure broad applicability. Although internal validation demonstrated consistent performance, multicenter external validation across heterogeneous health care systems is required to assess generalizability prior to clinical implementation. Prospective studies are also needed to evaluate clinical use and real-world effectiveness in live diagnostic workflows. Incorporating more robust missing data handling strategies, such as iterative imputation (eg, Multivariate Imputation by Chained Equations [56]), could more dynamically capture physiological dependencies among continuous clinical variables. Furthermore, integrating longitudinal patient data may allow the model to provide dynamic risk assessments as new clinical information becomes available over time. Additional research could explore deeper integration with imaging modalities, including advanced radiomic features or the direct use of raw imaging data, potentially enhancing diagnostic accuracy. Finally, improving scalability and computational efficiency through model compression techniques or cloud-based solutions could facilitate wider clinical adoption in resource-constrained clinical settings.

### Conclusions

This study developed and evaluated a multimodal malignancy prediction pipeline that integrates structured EHR variables with radiology report–derived features, including LLM-extracted abnormality characteristics and transformer-based embeddings of kidney-specific findings. Across systematic comparisons of early, middle, and late fusion, multimodal models achieved higher descriptive performance than unimodal baselines, with early fusion yielding the highest AUC and *F*_1_-score. Ablation and interpretability analyses indicated that structured clinical variables and kidney-specific report embeddings appeared to provide complementary predictive value: clinical variables provided the strongest signal in the early fusion setting, whereas kidney-specific text embeddings contributed most strongly in the middle and late fusion models. Together, these findings suggest that multimodal EHR modeling is a promising and scalable approach for supporting preoperative renal mass risk stratification, although external validation and formal statistical comparisons are needed before claims of superiority or clinical effectiveness can be made.

## Data Availability

The datasets generated during and/or analyzed during this study are not publicly available due to patient confidentiality constraints and institutional review board restrictions, but are available from the corresponding author on reasonable request and with approval from the institutional review committee.

## Appendix

Multimedia Appendix 1 Supplementary tables and figures.

## Abbreviations

AdamW: Adam optimizer with decoupled weight decay
AUC: area under the receiver operating characteristic curve
BERT: Bidirectional Encoder Representations from Transformers
BioBERT: biomedical BERT
BLEU-4: Bilingual Evaluation Understudy-4
ClinicalBERT: BERT pretrained on clinical text
CT: computed tomography
EHR: electronic health record
ICD: International Classification of Diseases
KC: kidney cancer
LLM: large language model
MLP: multilayer perceptron
NLP: natural language processing
OOF: out-of-fold
PheWAS: Phenome-Wide Association Study
PubMedBERT: BERT pretrained on PubMed text
RadBERT: radiology BERT
RCC: renal cell carcinoma
RF: random forest
SHAP: Shapley additive explanations
SVM: support vector machine
