# Supplementary material for: Multimodal Prediction of Renal Tumor Malignancy From Radiology Reports and Structured Electronic Health Records: Retrospective Cohort Study
Source: JMIR Med Inform. 2026 May 27;14:e84396. doi: 10.2196/84396 (PMC13254510; doi:10.2196/84396)

**Appendix Online Content**

This appendix file contains the additional tables of the experimental results for readers. The content is outlined as follows:

- Table s1. Cohort Characteristics
- Table s2. Performance Metrics of Clinical Variables Baseline Models for Renal Tumor Prediction
- Table s3. Performance Metrics of Abnormality Characteristics Baseline Models for Renal Tumor Prediction
- Table s4. Performance Metrics of Early Fusion Using Different BERT Encoders and Modality Exclusion for Renal Tumor Prediction
- Table s5. Performance Metrics of Middle Fusion Using Different BERT Encoders and Modality Exclusion for Renal Tumor Prediction
- Table s6. Performance Metrics of Three-Modality Late Fusion with Different Pretrained BERT and Baseline Model Combinations for Renal Tumor Prediction
- Table s7. Feature Engineering Summary and Missing Data Handling
- Table s8. Missingness Percentages of Continuous Clinical Variables (Vital Signs and Laboratory Measurements)
- Figure s9. Temporal Relationship Between Imaging, Diagnosis, Outcome Labeling, and Surgical Intervention
- Table s10. Inter-rater Agreement for Abnormality Attributes (Cohen’s κ)
- Figure s11. Top 15 Words by Average Layer Integrated Gradients Contribution Score in The Text-Modality Model.

**Table s1. Cohort Characteristics**

|  | UF Health  (N =967) |
| --- | --- |
| **Age**, mean (std) | 69.18 (12.93) |
| **Gender**, N (%) |  |
| Male | 568 (58.74%) |
| Female | 399 (41.26%) |
| **Race**, N (%) |  |
| White | 690 (71.35%) |
| Black or African American | 200 (20.68%) |
| Hispanic | 43 (4.45%) |
| Other | 10 (1.03%) |
| Unknown | 24 (2.48%) |
| **BMI**, mean (std) | 30.07 (7.08) |
| **Smoking**, N (%) | 150 (15.51%) |
| **Malignancy**, N (%) | 712 (73.63%) |
| **Nephrectomy**, N (%) | 401 (41.47%) |
| **Top 10 Diseases**, N (%) |  |
| Other disorders of the kidney and ureters | 874 (90.38%) |
| Hypertension | 719 (74.35%) |
| Disorders of lipoid metabolism | 525 (54.29%) |
| Other symptoms of respiratory system | 443 (45.81%) |
| Diseases of esophagus | 429 (44.36%) |
| Renal failure | 407 (42.09%) |
| Diabetes mellitus | 405 (41.88%) |
| Cardiac dysrhythmias | 385 (39.81%) |
| Abdominal pain | 370 (38.26%) |
| Pain in joint | 366 (37.85%) |
| **Top 10 Drug Usages**, N (%) |  |
| X-ray contrast media, iodinated | 888 (91.83%) |
| Other analgesics and antipyretics | 833 (86.14%) |
| Irrigating solutions | 824 (85.21%) |
| I.v. solution additives | 822 (85.01%) |
| Other mineral supplements | 822 (85.01%) |
| Opioids | 793 (82.01%) |
| Antiemetics and antinauseants | 762 (78.80%) |
| Agents for treatment of hemorrhoids and anal fissures for topical use | 754 (77.97%) |
| Antipruritics, incl. antihistamines, anesthetics, etc. | 751 (77.66%) |
| Antithrombotic agents | 726 (75.08%) |

**Table s2. Performance Metrics of Clinical Variables Baseline Models for Renal Tumor Prediction**

| **Model*** | **Overall Accuracy** | **Balanced Accuracy** | **Sensitivity** | **Specificity** | **Precision** | **F1** | **AUROC** |
| --- | --- | --- | --- | --- | --- | --- | --- |
| **LASSO (Over-sampling)** | 0.729 (± 0.026) | 0.706 (± 0.003) | 0.754 (± 0.053) | 0.659 (± 0.051) | 0.862 (± 0.009) | 0.803 (± 0.026) | 0.750 (± 0.006) |
| **LASSO (Under-sampling)** | 0.661 (± 0.033) | 0.717 (± 0.018) | 0.599 (± 0.059) | 0.835 (± 0.055) | 0.912 (± 0.020) | 0.721 (± 0.040) | 0.747 (± 0.010) |
| **LASSO (Baseline)** | 0.666 (± 0.146) | 0.689 (± 0.076) | 0.641 (± 0.226) | 0.737 (± 0.084) | 0.868 (± 0.023) | 0.717 (± 0.191) | 0.698 (± 0.125) |
| **LR (Over-sampling)** | 0.693 (± 0.020) | 0.681 (± 0.024) | 0.706 (± 0.047) | 0.655 (± 0.082) | 0.853 (± 0.024) | 0.772 (± 0.022) | 0.711 (± 0.027) |
| **LR (Under-sampling)** | 0.674 (± 0.047) | 0.707 (± 0.034) | 0.638 (± 0.066) | 0.776 (± 0.041) | 0.889 (± 0.018) | 0.741 (± 0.049) | 0.728 (± 0.043) |
| **LR (Baseline)** | 0.661 (± 0.062) | 0.684 (± 0.029) | 0.635 (± 0.099) | 0.733 (± 0.043) | 0.870 (± 0.004) | 0.730 (± 0.071) | 0.704 (± 0.039) |
| **MLP (Over-sampling)** | 0.712 (± 0.034) | 0.635 (± 0.004) | 0.799 (± 0.068) | 0.471 (± 0.060) | 0.809 (± 0.004) | 0.803 (± 0.031) | 0.631 (± 0.013) |
| **MLP (Under-sampling)** | 0.626 (± 0.101) | 0.646 (± 0.074) | 0.603 (± 0.168) | 0.690 (± 0.175) | 0.854 (± 0.064) | 0.693 (± 0.115) | 0.641 (± 0.103) |
| **MLP (Baseline)** | 0.618 (± 0.200) | 0.661 (± 0.090) | 0.569 (± 0.322) | 0.753 (± 0.145) | 0.693 (± 0.388) | 0.624 (± 0.350) | 0.670 (± 0.151) |
| **RF (Over-sampling)** | 0.755 (± 0.025) | 0.690 (± 0.011) | 0.827 (± 0.041) | 0.553 (± 0.026) | 0.838 (± 0.003) | 0.832 (± 0.021) | 0.728 (± 0.019) |
| **RF (Under-sampling)** | 0.761 (± 0.032) | 0.710 (± 0.013) | 0.817 (± 0.058) | 0.604 (± 0.047) | 0.853 (± 0.008) | 0.833 (± 0.029) | 0.754 (± 0.006) |
| **RF (Baseline)** | 0.741 (± 0.047) | 0.684 (± 0.029) | 0.804 (± 0.071) | 0.565 (± 0.035) | 0.838 (± 0.011) | 0.820 (± 0.041) | 0.724 (± 0.033) |
| **SVM (Over-sampling)** | 0.691 (± 0.037) | 0.649 (± 0.004) | 0.737 (± 0.077) | 0.561 (± 0.077) | 0.826 (± 0.011) | 0.777 (± 0.041) | 0.658 (± 0.013) |
| **SVM (Under-sampling)** | 0.712 (± 0.038) | 0.717 (± 0.016) | 0.708 (± 0.072) | 0.725 (± 0.071) | 0.880 (± 0.019) | 0.782 (± 0.037) | 0.758 (± 0.012) |
| **SVM (Baseline)** | 0.612 (± 0.196) | 0.649 (± 0.088) | 0.572 (± 0.322) | 0.725 (± 0.179) | 0.685 (± 0.384) | 0.622 (± 0.348) | 0.680 (± 0.103) |
| **XGBoost (Over-sampling)** | 0.686 (± 0.046) | 0.685 (± 0.033) | 0.687 (± 0.070) | 0.682 (± 0.063) | 0.859 (± 0.021) | 0.761 (± 0.045) | 0.724 (± 0.036) |
| **XGBoost (Under-sampling)** | 0.685 (± 0.063) | 0.707 (± 0.037) | 0.660 (± 0.110) | 0.753 (± 0.105) | 0.886 (± 0.038) | 0.751 (± 0.065) | 0.725 (± 0.044) |
| **XGBoost (Baseline)** | 0.693 (± 0.049) | 0.669 (± 0.011) | 0.719 (± 0.099) | 0.620 (± 0.094) | 0.843 (± 0.015) | 0.773 (± 0.048) | 0.688 (± 0.019) |

***Model abbreviations:** LASSO = Least Absolute Shrinkage and Selection Operator Regression; LR = Logistic Regression; MLP = Multilayer Perceptron; RF = Random Forest; SVM = Support Vector Machine; XGBoost = eXtreme Gradient Boosting.

**Table s3. Performance Metrics of Abnormality Characteristics Baseline Models for Renal Tumor Prediction**

| **Model*** | **Overall Accuracy** | **Balanced Accuracy** | **Sensitivity** | **Specificity** | **Precision** | **F1** | **AUROC** |
| --- | --- | --- | --- | --- | --- | --- | --- |
| **LASSO (Over-sampling)** | 0.667 (± 0.048) | 0.680 (± 0.019) | 0.653 (± 0.115) | 0.706 (± 0.144) | 0.868 (± 0.036) | 0.739 (± 0.060) | 0.711 (± 0.018) |
| **LASSO (Under-sampling)** | 0.525 (± 0.239) | 0.607 (± 0.098) | 0.434 (± 0.396) | 0.780 (± 0.201) | 0.508 (± 0.464) | 0.468 (± 0.427) | 0.608 (± 0.099) |
| **LASSO (Baseline)** | 0.546 (± 0.158) | 0.660 (± 0.089) | 0.421 (± 0.235) | 0.898 (± 0.059) | 0.737 (± 0.412) | 0.536 (± 0.299) | 0.671 (± 0.096) |
| **LR (Over-sampling)** | 0.691 (± 0.040) | 0.684 (± 0.010) | 0.698 (± 0.094) | 0.671 (± 0.114) | 0.860 (± 0.028) | 0.766 (± 0.048) | 0.716 (± 0.015) |
| **LR (Under-sampling)** | 0.686 (± 0.016) | 0.683 (± 0.011) | 0.688 (± 0.038) | 0.678 (± 0.051) | 0.858 (± 0.013) | 0.763 (± 0.019) | 0.682 (± 0.020) |
| **LR (Baseline)** | 0.679 (± 0.009) | 0.701 (± 0.023) | 0.656 (± 0.028) | 0.745 (± 0.069) | 0.880 (± 0.024) | 0.751 (± 0.011) | 0.709 (± 0.015) |
| **MLP (Over-sampling)** | 0.680 (± 0.102) | 0.581 (± 0.008) | 0.790 (± 0.219) | 0.373 (± 0.226) | 0.783 (± 0.017) | 0.772 (± 0.108) | 0.571 (± 0.020) |
| **MLP (Under-sampling)** | 0.661 (± 0.061) | 0.648 (± 0.058) | 0.676 (± 0.103) | 0.620 (± 0.144) | 0.835 (± 0.044) | 0.743 (± 0.063) | 0.644 (± 0.055) |
| **MLP (Baseline)** | 0.682 (± 0.028) | 0.677 (± 0.030) | 0.688 (± 0.085) | 0.667 (± 0.139) | 0.857 (± 0.033) | 0.760 (± 0.037) | 0.697 (± 0.022) |
| **RF (Over-sampling)** | 0.655 (± 0.019) | 0.618 (± 0.014) | 0.695 (± 0.038) | 0.541 (± 0.049) | 0.810 (± 0.011) | 0.747 (± 0.021) | 0.627 (± 0.014) |
| **RF (Under-sampling)** | 0.646 (± 0.039) | 0.678 (± 0.030) | 0.611 (± 0.060) | 0.745 (± 0.059) | 0.871 (± 0.023) | 0.717 (± 0.041) | 0.676 (± 0.018) |
| **RF (Baseline)** | 0.642 (± 0.020) | 0.677 (± 0.020) | 0.604 (± 0.032) | 0.749 (± 0.047) | 0.871 (± 0.018) | 0.713 (± 0.021) | 0.670 (± 0.007) |
| **SVM (Over-sampling)** | 0.692 (± 0.023) | 0.679 (± 0.007) | 0.706 (± 0.049) | 0.651 (± 0.053) | 0.851 (± 0.012) | 0.771 (± 0.026) | 0.688 (± 0.018) |
| **SVM (Under-sampling)** | 0.710 (± 0.007) | 0.662 (± 0.023) | 0.764 (± 0.036) | 0.561 (± 0.082) | 0.831 (± 0.022) | 0.795 (± 0.012) | 0.657 (± 0.017) |
| **SVM (Baseline)** | 0.716 (± 0.000) | 0.656 (± 0.000) | 0.783 (± 0.000) | 0.529 (± 0.000) | 0.824 (± 0.000) | 0.803 (± 0.000) | 0.643 (± 0.002) |
| **XGBoost (Over-sampling)** | 0.605 (± 0.113) | 0.597 (± 0.022) | 0.614 (± 0.224) | 0.580 (± 0.202) | 0.820 (± 0.052) | 0.672 (± 0.181) | 0.601 (± 0.018) |
| **XGBoost (Under-sampling)** | 0.602 (± 0.073) | 0.633 (± 0.030) | 0.568 (± 0.152) | 0.698 (± 0.164) | 0.851 (± 0.051) | 0.668 (± 0.100) | 0.635 (± 0.024) |
| **XGBoost (Baseline)** | 0.634 (± 0.065) | 0.655 (± 0.021) | 0.611 (± 0.134) | 0.698 (± 0.138) | 0.857 (± 0.036) | 0.704 (± 0.087) | 0.663 (± 0.039) |

***Model abbreviations:** LASSO = Least Absolute Shrinkage and Selection Operator Regression; LR = Logistic Regression; MLP = Multilayer Perceptron; RF = Random Forest; SVM = Support Vector Machine; XGBoost = eXtreme Gradient Boosting.

**Table s4. Performance Metrics of Early Fusion Using Different BERT Encoders and Modality Exclusion for Renal Tumor Prediction**

[Alt text: Table s4 BERT Encoders mainly using to handle KF (Kidney-specific findings), input modalities denoted as KF (Kidney-specific findings), AC (Abnormality characteristics), and CV (Clinical variables).]

| **Model** | **Overall Accuracy** | **Balanced Accuracy** | **Sensitivity** | **Specificity** | **Precision** | **F1** | **AUROC** |
| --- | --- | --- | --- | --- | --- | --- | --- |
| **RadBERT** |  |  |  |  |  |  |  |
| **KF + AC + CV** | 0.750 (±0.029) | 0.775 (±0.010) | 0.722 (±0.062) | 0.828 (±0.069) | 0.924 (±0.021) | 0.809 (±0.030) | 0.813 (±0.008) |
| **KF + CV** | 0.749 (±0.046) | 0.759 (±0.030) | 0.738 (±0.087) | 0.780 (±0.097) | 0.907 (±0.032) | 0.811 (±0.049) | 0.796 (±0.030) |
| **KF + AC** | 0.687 (±0.041) | 0.727 (±0.037) | 0.642 (±0.048) | 0.812 (±0.041) | 0.905 (±0.023) | 0.751 (±0.038) | 0.755 (±0.061) |
| **AC + CV** | 0.763 (±0.052) | 0.743 (±0.010) | 0.785 (±0.108) | 0.701 (±0.108) | 0.884 (±0.027) | 0.827 (±0.051) | 0.779 (±0.010) |
| **ClinicalBERT** |  |  |  |  |  |  |  |
| **KF + AC + CV** | 0.637 (±0.208) | 0.706 (±0.115) | 0.561 (±0.312) | 0.851 (±0.088) | 0.930 (±0.041) | 0.637 (±0.343) | 0.750 (±0.167) |
| **KF + CV** | 0.661 (±0.226) | 0.717 (±0.123) | 0.599 (±0.343) | 0.835 (±0.120) | 0.930 (±0.045) | 0.657 (±0.363) | 0.726 (±0.204) |
| **KF + AC** | 0.679 (±0.048) | 0.693 (±0.057) | 0.664 (±0.081) | 0.722 (±0.142) | 0.874 (±0.051) | 0.752 (±0.046) | 0.729 (±0.091) |
| **AC + CV** | 0.763 (±0.052) | 0.743 (±0.010) | 0.785 (±0.108) | 0.701 (±0.108) | 0.884 (±0.027) | 0.827 (±0.051) | 0.779 (±0.010) |
| **PubMedBERT** |  |  |  |  |  |  |  |
| **KF + AC + CV** | 0.701 (±0.120) | 0.674 (±0.085) | 0.731 (±0.170) | 0.616 (±0.101) | 0.838 (±0.046) | 0.774 (±0.121) | 0.697 (±0.117) |
| **KF + CV** | 0.654 (±0.219) | 0.654 (±0.090) | 0.653 (±0.366) | 0.655 (±0.201) | 0.673 (±0.377) | 0.663 (±0.371) | 0.658 (±0.156) |
| **KF + AC** | 0.598 (±0.067) | 0.659 (±0.043) | 0.530 (±0.100) | 0.788 (±0.061) | 0.875 (±0.030) | 0.656 (±0.073) | 0.666 (±0.056) |
| **AC + CV** | 0.763 (±0.052) | 0.743 (±0.010) | 0.785 (±0.108) | 0.701 (±0.108) | 0.884 (±0.027) | 0.827 (±0.051) | 0.779 (±0.010) |
| **BioBERT** |  |  |  |  |  |  |  |
| **KF + AC + CV** | 0.631 (±0.143) | 0.689 (±0.100) | 0.566 (±0.205) | 0.812 (±0.132) | 0.887 (±0.084) | 0.676 (±0.172) | 0.724 (±0.148) |
| **KF + CV** | 0.601 (±0.190) | 0.680 (±0.105) | 0.513 (±0.288) | 0.847 (±0.110) | 0.724 (±0.406) | 0.600 (±0.336) | 0.686 (±0.188) |
| **KF + AC** | 0.701 (±0.057) | 0.684 (±0.052) | 0.720 (±0.088) | 0.647 (±0.113) | 0.853 (±0.039) | 0.778 (±0.052) | 0.696 (±0.058) |
| **AC + CV** | 0.763 (±0.052) | 0.743 (±0.010) | 0.785 (±0.108) | 0.701 (±0.108) | 0.884 (±0.027) | 0.827 (±0.051) | 0.779 (±0.010) |

**Table s5. Performance Metrics of Middle Fusion Using Different BERT Encoders and Modality Exclusion for Renal Tumor Prediction**

[Alt text: Table s5 BERT Encoders mainly using to handle KF (Kidney-specific findings), input modalities denoted as KF (Kidney-specific findings), AC (Abnormality characteristics), and CV (Clinical variables).]

| **Model** | **Overall Accuracy** | **Balanced Accuracy** | **Sensitivity** | **Specificity** | **Precision** | **F1** | **AUROC** |
| --- | --- | --- | --- | --- | --- | --- | --- |
| **RadBERT** |  |  |  |  |  |  |  |
| **KF + AC + CV** | 0.726 (±0.028) | 0.744 (±0.018) | 0.705 (±0.040) | 0.784 (±0.016) | 0.902 (±0.006) | 0.791 (±0.026) | 0.782 (±0.011) |
| **KF + CV** | 0.699 (±0.069) | 0.730 (±0.059) | 0.664 (±0.083) | 0.796 (±0.045) | 0.899 (±0.032) | 0.763 (±0.066) | 0.749 (±0.074) |
| **KF + AC** | 0.670 (±0.056) | 0.711 (±0.043) | 0.625 (±0.081) | 0.796 (±0.069) | 0.897 (±0.033) | 0.734 (±0.058) | 0.747 (±0.052) |
| **AC + CV** | 0.715 (±0.060) | 0.698 (±0.030) | 0.734 (±0.118) | 0.662 (±0.124) | 0.862 (±0.027) | 0.788 (±0.063) | 0.731 (±0.019) |
| **ClinicalBERT** |  |  |  |  |  |  |  |
| **KF + AC + CV** | 0.653 (±0.219) | 0.709 (±0.118) | 0.590 (±0.333) | 0.827 (±0.114) | 0.725 (±0.406) | 0.650 (±0.364) | 0.713 (±0.204) |
| **KF + CV** | 0.642 (±0.185) | 0.709 (±0.107) | 0.568 (±0.273) | 0.851 (±0.067) | 0.904 (±0.029) | 0.659 (±0.284) | 0.735 (±0.172) |
| **KF + AC** | 0.704 (±0.087) | 0.710 (±0.056) | 0.698 (±0.129) | 0.722 (±0.075) | 0.875 (±0.030) | 0.771 (±0.090) | 0.754 (±0.082) |
| **AC + CV** | 0.715 (±0.060) | 0.698 (±0.030) | 0.734 (±0.118) | 0.662 (±0.124) | 0.862 (±0.027) | 0.788 (±0.063) | 0.731 (±0.019) |
| **PubMedBERT** |  |  |  |  |  |  |  |
| **KF + AC + CV** | 0.558 (±0.222) | 0.624 (±0.086) | 0.484 (±0.375) | 0.765 (±0.208) | 0.878 (±0.040) | 0.536 (±0.356) | 0.629 (±0.134) |
| **KF + CV** | 0.516 (±0.167) | 0.603 (±0.083) | 0.421 (±0.269) | 0.784 (±0.145) | 0.872 (±0.087) | 0.514 (±0.295) | 0.581 (±0.157) |
| **KF + AC** | 0.600 (±0.078) | 0.650 (±0.035) | 0.544 (±0.128) | 0.757 (±0.069) | 0.864 (±0.014) | 0.660 (±0.100) | 0.652 (±0.055) |
| **AC + CV** | 0.715 (±0.060) | 0.698 (±0.030) | 0.734 (±0.118) | 0.662 (±0.124) | 0.862 (±0.027) | 0.788 (±0.063) | 0.731 (±0.019) |
| **BioBERT** |  |  |  |  |  |  |  |
| **KF + AC + CV** | 0.601 (±0.194) | 0.645 (±0.083) | 0.552 (±0.323) | 0.737 (±0.181) | 0.687 (±0.385) | 0.608 (±0.344) | 0.650 (±0.168) |
| **KF + CV** | 0.637 (±0.105) | 0.668 (±0.061) | 0.603 (±0.156) | 0.733 (±0.063) | 0.861 (±0.028) | 0.700 (±0.125) | 0.699 (±0.091) |
| **KF + AC** | 0.725 (±0.056) | 0.682 (±0.024) | 0.772 (±0.102) | 0.592 (±0.085) | 0.842 (±0.014) | 0.802 (±0.054) | 0.708 (±0.022) |
| **AC + CV** | 0.715 (±0.060) | 0.698 (±0.030) | 0.734 (±0.118) | 0.662 (±0.124) | 0.862 (±0.027) | 0.788 (±0.063) | 0.731 (±0.019) |

**Table s6. Performance Metrics of Three-Modality Late Fusion with Different Pretrained BERT and Baseline Model Combinations for Renal Tumor Prediction**

| **Model** | **Overall Accuracy** | **Balanced Accuracy** | **Sensitivity** | **Specificity** | **Precision** | **F1** | **AUROC** |
| --- | --- | --- | --- | --- | --- | --- | --- |
| **RadBERT_LASSO** | 0.737 (±0.053) | 0.767 (±0.017) | 0.703 (±0.094) | 0.831 (±0.060) | 0.923 (±0.018) | 0.795 (±0.057) | 0.799 (±0.020) |
| **RadBERT_LR** | 0.723 (±0.034) | 0.744 (±0.033) | 0.699 (±0.040) | 0.788 (±0.047) | 0.903 (±0.022) | 0.788 (±0.030) | 0.778 (±0.024) |
| **RadBERT_MLP** | 0.741 (±0.053) | 0.761 (±0.027) | 0.719 (±0.083) | 0.804 (±0.039) | 0.912 (±0.010) | 0.802 (±0.051) | 0.797 (±0.017) |
| **RadBERT_RF** | 0.736 (±0.021) | 0.749 (±0.013) | 0.722 (±0.053) | 0.776 (±0.072) | 0.902 (±0.022) | 0.800 (±0.025) | 0.805 (±0.016) |
| **RadBERT_XGBoost** | 0.744 (±0.040) | 0.745 (±0.036) | 0.744 (±0.048) | 0.745 (±0.042) | 0.891 (±0.019) | 0.810 (±0.033) | 0.788 (±0.033) |
| **RadBERT_SVM** | 0.698 (±0.069) | 0.704 (±0.015) | 0.691 (±0.142) | 0.718 (±0.142) | 0.880 (±0.037) | 0.764 (±0.080) | 0.754 (±0.020) |
| **PubMedBERT_ LASSO** | 0.686 (±0.040) | 0.701 (±0.062) | 0.669 (±0.043) | 0.733 (±0.127) | 0.878 (±0.046) | 0.758 (±0.032) | 0.740 (±0.077) |
| **PubMedBERT_LR** | 0.663 (±0.101) | 0.659 (±0.029) | 0.667 (±0.211) | 0.651 (±0.221) | 0.858 (±0.058) | 0.729 (±0.122) | 0.688 (±0.043) |
| **PubMedBERT_MLP** | 0.670 (±0.048) | 0.711 (±0.035) | 0.625 (±0.083) | 0.796 (±0.090) | 0.898 (±0.032) | 0.734 (±0.053) | 0.742 (±0.043) |
| **PubMedBERT_RF** | 0.674 (±0.027) | 0.705 (±0.016) | 0.641 (±0.049) | 0.769 (±0.049) | 0.887 (±0.015) | 0.743 (±0.031) | 0.756 (±0.016) |
| **PubMedBERT_XGBoost** | 0.658 (±0.055) | 0.688 (±0.015) | 0.624 (±0.110) | 0.753 (±0.105) | 0.881 (±0.030) | 0.724 (±0.069) | 0.729 (±0.025) |
| **PubMedBERT_SVM** | 0.691 (±0.024) | 0.658 (±0.012) | 0.727 (±0.043) | 0.588 (±0.039) | 0.832 (±0.008) | 0.776 (±0.024) | 0.664 (±0.045) |
| **ClinicalBERT_ LASSO** | 0.739 (±0.054) | 0.749 (±0.056) | 0.729 (±0.082) | 0.769 (±0.120) | 0.901 (±0.046) | 0.803 (±0.049) | 0.795 (±0.050) |
| **ClinicalBERT_LR** | 0.714 (±0.040) | 0.710 (±0.040) | 0.719 (±0.063) | 0.702 (±0.090) | 0.872 (±0.031) | 0.787 (±0.037) | 0.765 (±0.044) |
| **ClinicalBERT_MLP** | 0.713 (±0.039) | 0.739 (±0.038) | 0.685 (±0.078) | 0.792 (±0.116) | 0.907 (±0.040) | 0.777 (±0.043) | 0.786 (±0.038) |
| **ClinicalBERT_RF** | 0.728 (±0.025) | 0.746 (±0.025) | 0.708 (±0.071) | 0.784 (±0.113) | 0.906 (±0.040) | 0.792 (±0.032) | 0.797 (±0.025) |
| **ClinicalBERT_XGBoost** | 0.709 (±0.052) | 0.732 (±0.039) | 0.684 (±0.081) | 0.780 (±0.079) | 0.899 (±0.029) | 0.774 (±0.052) | 0.783 (±0.035) |
| **ClinicalBERT_SVM** | 0.692 (±0.059) | 0.701 (±0.020) | 0.681 (±0.109) | 0.722 (±0.087) | 0.875 (±0.020) | 0.761 (±0.067) | 0.746 (±0.022) |
| **BioBERT_ LASSO** | 0.657 (±0.070) | 0.713 (±0.034) | 0.594 (±0.112) | 0.831 (±0.051) | 0.910 (±0.013) | 0.713 (±0.086) | 0.754 (±0.056) |
| **BioBERT_LR** | 0.695 (±0.024) | 0.682 (±0.014) | 0.709 (±0.046) | 0.655 (±0.049) | 0.853 (±0.013) | 0.773 (±0.024) | 0.711 (±0.034) |
| **BioBERT_MLP** | 0.687 (±0.054) | 0.718 (±0.020) | 0.652 (±0.105) | 0.784 (±0.101) | 0.899 (±0.035) | 0.750 (±0.064) | 0.757 (±0.023) |
| **BioBERT_RF** | 0.676 (±0.084) | 0.707 (±0.031) | 0.642 (±0.143) | 0.773 (±0.086) | 0.891 (±0.017) | 0.737 (±0.097) | 0.760 (±0.029) |
| **BioBERT_XGBoost** | 0.675 (±0.060) | 0.699 (±0.030) | 0.649 (±0.105) | 0.749 (±0.089) | 0.881 (±0.026) | 0.743 (±0.069) | 0.743 (±0.033) |
| **BioBERT_SVM** | 0.733 (±0.023) | 0.676 (±0.025) | 0.796 (±0.047) | 0.557 (±0.077) | 0.835 (±0.020) | 0.814 (±0.021) | 0.696 (±0.035) |

***Model abbreviations:** LASSO = Least Absolute Shrinkage and Selection Operator Regression; LR = Logistic Regression; MLP = Multilayer Perceptron; RF = Random Forest; SVM = Support Vector Machine; XGBoost = eXtreme Gradient Boosting.

**Table s7. Feature Engineering Summary and Missing Data Handling**

| **Feature modality** | **Feature type** | **Examples** | **No. of features** | **Missingness handling** | **Dimensionality** |
| --- | --- | --- | --- | --- | --- |
| **Structured clinical variables (EHR)** | Demographics, diagnoses, medications, vitals, labs | Age, sex, Phecodes, ATC drug indicators, blood pressure, labs | 745 | Continuous variables imputed using cohort mean; categorical variables binarized with missing encoded as 0 (absence) | 765 |
| **Abnormality characteristics (radiology-derived)** | Categorical | Abnormality type, position, exophytic status, attenuation, enhancement, lesion type | 12 | Filled as “unknown” when not explicitly mentioned; one-hot encoded | 53 |
| **Abnormality characteristics (radiology-derived)** | Continuous | Lesion size (cm), tumor growth rate (TGR) | 2 | Sentinel handling followed by zero-imputation for non-mentioned or unavailable values | 4 |
| **Kidney-specific findings (text)** | Transformer embeddings | Kidney-related CT report paragraphs | 1 paragraph text | Not applicable | 768 |

**Table s8. Missingness Percentages of Continuous Clinical Variables (Vital Signs and Laboratory Measurements)**

| **Clinical Variables** | **Missing Percentage** |
| --- | --- |
| **Vital Signs** |  |
| Highest SBP (most recent day) | 0.00% |
| Lowest SBP (most recent day) | 0.00% |
| Highest DBP (most recent day) | 0.00% |
| Lowest DBP (most recent day) | 0.00% |
| BMI | 4.03% |
| **Laboratory Measurements** |  |
| Creatinine | 4.45% |
| Glucose | 4.45% |
| Blood Urea Nitrogen | 4.55% |
| Calcium | 4.55% |
| Sodium | 4.55% |
| Chloride | 4.65% |
| Potassium | 4.65% |
| Red blood cell count | 6.20% |
| Hemoglobin | 6.20% |
| Mean Corpuscular Hemoglobin (MCH) in Red Blood Cells by count | 6.31% |
| Mean Corpuscular Hemoglobin Concentration (MCHC) in Red Blood Cells by count | 6.31% |
| Mean Platelet Volume (MPV) | 9.41% |
| Monocytes | 16.55% |
| Neutrophils | 16.65% |
| Eosinophils | 16.86% |
| Total Protein | 19.54% |
| ALP | 19.75% |
| AST | 19.96% |
| Albumin | 19.96% |
| ALT | 20.68% |
| Bilirubin, total | 20.68% |
| Basophils | 25.03% |
| Mean Corpuscular Volume (MCV) in Red Blood Cells by count | 25.96% |
| Red Cell Distribution Width (RDW) | 32.78% |
| BUN/Creatinine | 35.57% |
| Lymphocytes | 36.30% |
| HbA1C | 47.57% |
| HDL Cholesterol | 49.43% |
| Cholesterol | 49.53% |

**Figure s9. Temporal Relationship Between Imaging, Diagnosis, Outcome Labeling, and Surgical Intervention**
This schematic illustrates the temporal constraints applied in the study design. For each patient, only renal CT reports acquired prior to the index diagnosis date were used for feature extraction, representing pre-operative diagnostic assessment. Structured EHR variables were similarly restricted to the pre-index observation window. Final tumor status was determined using longitudinal diagnosis codes and could occur after the index date. Surgical interventions, when present, occurred downstream of both imaging and index diagnosis and did not contribute to model inputs, thereby preventing data leakage.


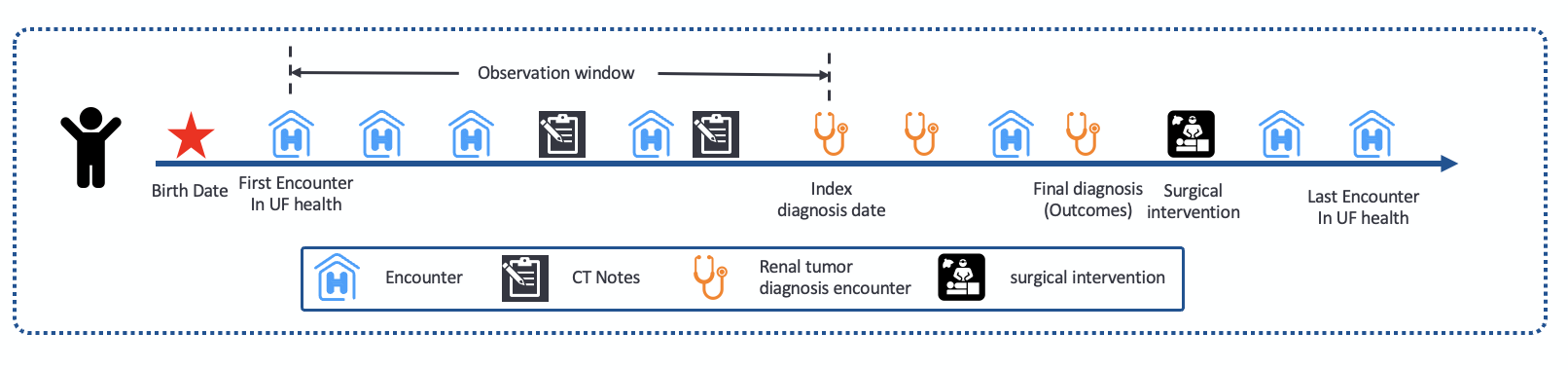


**Table s10. Inter-rater Agreement for Abnormality Attributes (Cohen’s κ)**

| **Abnormality Characteristics**^a^ | **Inter-rater Agreement**  **(Cohen’s κ)** |
| --- | --- |
| **Abnormality Presence** | 1.000 |
| **Abnormality Category** | 1.000 |
| **Position** | 0.992 |
| **Size(cm)** | 0.997 |
| **Exophytic** | 1.000 |
| **Attenuation** | 0.975 |
| **Enhancement** | 0.950 |

^a^Abnormality characteristics included abnormality presence, type (e.g., cyst, mass, tumor), lesion size, anatomical position (e.g., left kidney or right kidney), exophytic status, CT attenuation, and contrast enhancement.

**Figure s11. Top 15 Words by Average Layer Integrated Gradients Contribution Score in The Text-Modality Model**


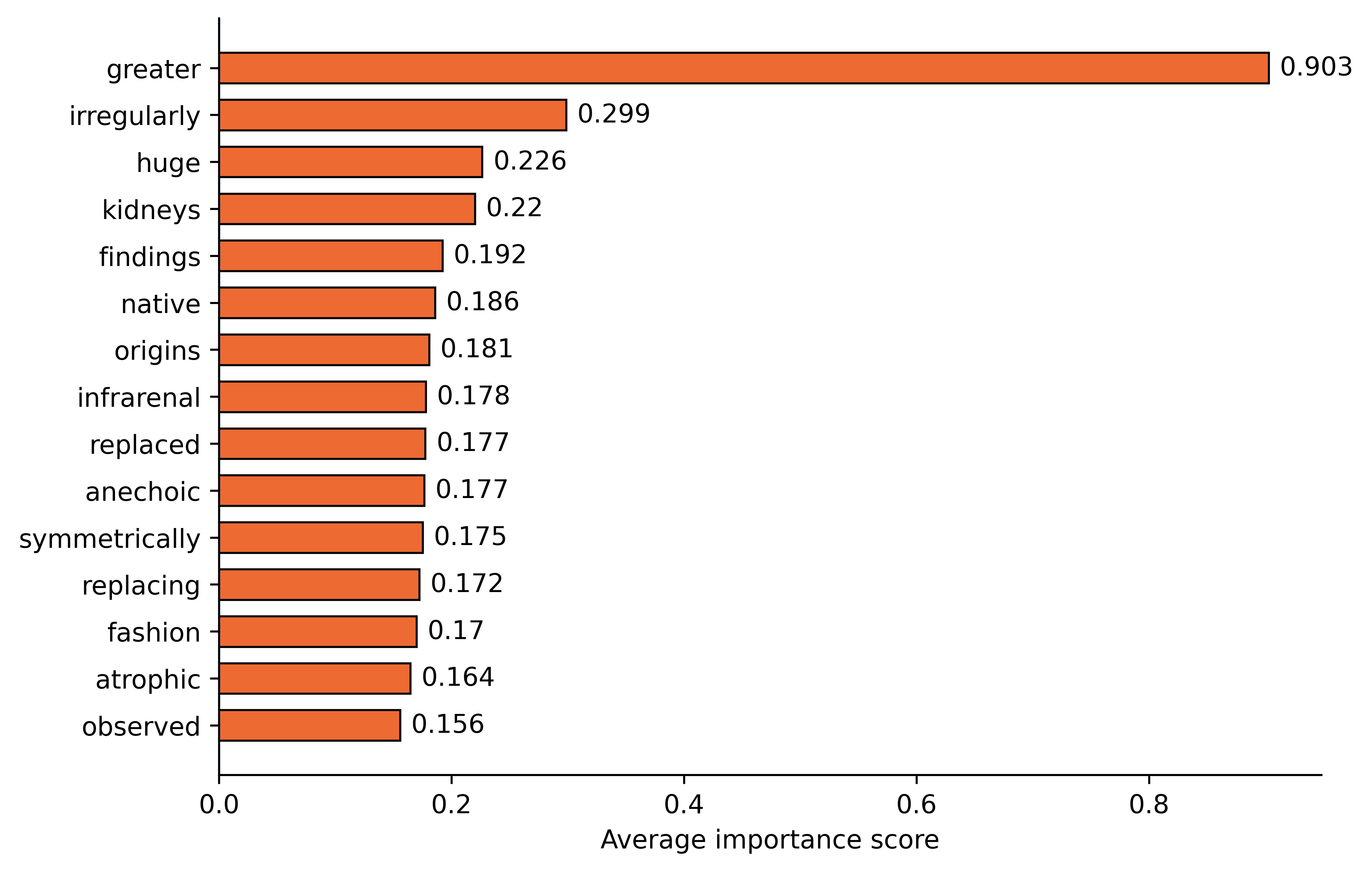

Supplement: Multimedia Appendix 1 [file medinform_v14i1e84396_app1.docx]
